# Exploring differences in protein cargo of extracellular vesicles from ME/CFS patient plasma compared to healthy controls

**DOI:** 10.1016/j.bbrep.2026.102679

**Published:** 2026-06-20

**Authors:** Anne Rydland, Elena Støvring Yran, Tuula A. Nyman, Elin Bolle Strand, Anne-Marie Siebke Trøseid, Reidun Øvstebø, Fatima Heinicke, Benedicte A. Lie, Marte K. Viken

**Affiliations:** a Department of Medical Genetics, University of Oslo and Oslo University Hospital, Oslo, Norway; b Center for Treatment of Rheumatic and Musculoskeletal Diseases (REMEDY), Diakonhjemmet Hospital, Oslo, Norway; c Department of Immunology, University of Oslo and Oslo University Hospital, Oslo, Norway; d Department of Digital Health Research, and CFS/ME Senteret, Oslo University Hospital, Oslo, Norway; e The Blood Cell Research Group, Department of Medical Biochemistry, Oslo University Hospital, Ullevål, Oslo, Norway; f Oslo Centre for Biostatistics and Epidemiology, Department of Biostatistics, Institute of Basic Medical Sciences, University of Oslo, Oslo, Norway

## Abstract

Myalgic encephalomyelitis/chronic fatigue syndrome (ME/CFS) is a chronic and debilitating disease characterized by post-exertional malaise, fatigue and pain. Yet, its underlying biological mechanisms remain poorly understood. Extracellular vesicles (EVs) are nanoparticles carrying biological cargo and are involved in cell-cell communication. Plasma EVs reflect several disease states and may serve as minimally invasive biomarkers. In this exploratory study, we characterized the plasma EV profiles of ME/CFS patients (N = 49) and healthy controls (N = 50), by enriching for EVs by size-exclusion chromatography coupled to high-resolution quantitative proteomics. The ME/CFS patients had significantly higher concentrations of EVs than healthy controls. Among the 424 detected proteins included for analyses, 11 had different levels in EVs from ME/CFS patients. The ME/CFS associated EV proteins appear to mainly originate from erythroid cells, hepatocytes and plasma B cells, based on their tissue expression. Albeit differences in EV protein levels did not withstand correction for multiple testing, our study is the largest to date, thereby encouraging future investigations on the role of EV and its cargo in ME/CFS.

## Highlights

• Increased number of EVs was observed in plasma from ME/CFS patients.
• Minor EV protein cargo differences in ME/CFS patients compared to controls.
• Slightly altered EV protein profile implicated complement and coagulation pathways.
• Exploratory study.

## 1 Introduction

Myalgic encephalomyelitis/chronic fatigue syndrome (ME/CFS) is a chronic disease characterized by a variety of symptoms including, albeit not limited to, post-exertional malaise (PEM), profound fatigue and pain. ME/CFS patients often have reduced quality of life with around 25% of patients being housebound [1]. The disease is estimated to affect up to 1% of the population, depending on the diagnostic criteria being used as the inclusion and exclusion criteria differ [2,3]. Although all require fatigue, some like the Fukuda Criteria do not require PEM as a key symptom [4], in contrast to e.g. the Canadian consensus Criteria and International Consensus Criteria [5,6]. Biomarkers and insight into the pathogenic mechanisms of ME/CFS are therefore highly warranted.

Extracellular vesicles (EVs) have recently emerged as potential diagnostic markers. EVs are involved in cell communication via transfer of cargo, like proteins, nucleic acids and lipids, between cells. Several studies have characterized circulatory EVs in ME/CFS, but with diverse study designs and conflicting results. The ME/CFS patients included have been diagnosed according to the less stringent 1994 CDC/Fukuda definition. The most consistent findings are a higher concentration and smaller size of circulatory EVs observed in ME/CFS patients compared to healthy controls [7,8], while others have reported higher concentration without significant difference in EV size [9,10], and one study observed larger mean size EVs in ME/CFS compared to controls [11]. The study by Bonilla and colleagues specifically addressed surface markers of EVs with proposed B-cell and platelet origin and reported a correlation with disease severity, notwithstanding statistical correction [12].

Furthermore, some of the studies have also investigated the levels of various EV cargo in blood samples from ME/CFS patients compared to controls. Differences in levels of miRNA have been reported [7], and also in mitochondrial DNA before and after exercise [13]. EV proteins have also been assessed to some extent. Interestingly, EV cytokine levels have been reported to be differential between ME/CFS patients (N = 49) and controls (N = 49), albeit not statistically significant after correcting for multiple testing [11]. One study comprehensively profiled EV proteins using nano-liquid chromatography-tandem mass spectrometry (LC-MS/MS) and reported higher levels of actin network proteins in EVs from ME/CFS patients, however, less than 10 patients were investigated [10]. Exercise have also been reported to alter EV proteins differently in ME/CFS patients (N = 18) compare to controls (N = 17) [14].

The promising, albeit conflicting, results from previous studies could be due to limited samples sizes when assessing EV protein profiles. Therefore, we hypothesize that EV protein cargo is altered in ME/CFS patients, and we performed an explorative study of the global EV proteome. Specifically, we examined the EV concentration and proteome cargo in a cohort of ME/CFS patients (N = 49), all diagnosed according to the stringent Canadian criteria, with the aim to both verify previous findings and reveal new insights into ME/CFS associated alterations in EV proteins.

## 2 Materials and methods

We have submitted information from our experiments to the EV-track knowledgebase (EV-TRACK ID: EV250105) [15]. The study was conducted in accordance with the Minimal information for studies of extracellular vesicles guidelines provided by the International Society for Extracellular Vesicles [16].

### 2.1 Study population

Our study population consisted of 49 ME/CFS patients and 50 healthy controls recruited in 2013- May 2019 at the specialist healthcare service, CFS/ME center, Oslo University Hospital. Hence, all individuals are pre-Covid 19 pandemic. All patients were diagnosed according to the 2003 Canadian Consensus Criteria [6]. Patients and controls were matched for sex, age and body mass index (BMI), and 20 of the 50 controls have previously been part of a study on rheumatoid arthritis [17]. For the ME/CFS patients, we had information about disease duration, disease severity last 6 months prior to inclusion, measured by activity assessment in question no. 79 DePaul Symptom Questionnaire, autoimmune disease in first degree relatives and self-reported symptom start following infectious episode or vaccination.

Written informed consent was given by all study participants. The study was approved by the Norwegian National Health Authorities and Regional Ethics Committee (REK 2015/1547) and executed in adherence to the Declaration of Helsinki.

### 2.2 Plasma collection and processing

Peripheral blood was collected in Vacuette K_2_EDTA tubes and processed within 30 min. The samples were centrifuged at 1800 g for 15 min to remove cellular debris followed by 15,000 g for 15 min at 4°C within 10 min to generate platelet-poor plasma and eliminate large EVs. The platelet-poor plasma was aliquoted and directly frozen at −80°C until EV isolation.

### 2.3 EV enrichment

Plasma was thawed at 4°C, and EVs were isolated by size exclusion chromatography from 500 μl plasma using a qEV original 70 nm column (SP1, Izon Science, Oxford, UK). EVs were eluted in 500 μl 0.2 μm filtered phosphate-buffer saline (PBS) and EV enriched fractions 7-9 were pooled and aliquoted for downstream analyses. EV enriched samples were directly submitted to nanoparticle tracking analysis (NTA) and transmission electron microscopy (TEM), while aliquots for protein analyses were stored at −80°C prior to analysis.

### 2.4 Nanoparticle tracking analysis

Nanoparticle tracking analysis (NTA) was performed at the Department of Medical Biochemistry (Oslo University Hospital) on samples from 20 randomly selected ME/CFS patients and results for 20 healthy controls previously published was included [17]. EV enriched samples were diluted 1:100 in 0.02 freshly filtered PBS and injected into the Nanosight NS500 (Malvern Instruments Ltd) instrument in a constant flow. The samples were measured as 60 s videos in triplicate with settings adjusted to camera level 14 and detection threshold 4. Size and concentration were calculated using NTA Software 3.4 (Malvern).

### 2.5 Transmission electron microscopy

Immuno-TEM was performed on two pools of EV enriched material, one consisting of 5 ME/CFS patients and the other of 5 healthy controls. The EV enriched samples were dropped onto formvar/carbon coated grids and left for 10 min at room temperature. Then EVs were fixed in 4% formaldehyde and 0.1% glutaraldehyde for 15 min. Grids were washed with PBS, blocked for 10 min with 0.5% bovine serum albumin in PBS, incubated for 20 min with mouse anti-CD63 primary antibody, incubated 20 min with rabbit anti-mouse secondary antibody and, lastly, incubated with protein A-gold (10 nm) for 15 min. Two washing steps with PBS were performed between each antibody incubation. Next, grids were washed five times in PBS and five times in water and stained with 0.4% uranyl acetate and embedded in 1.8% methyl cellulose for 10 min at 4°C. Grids were dried for 20 min and samples were investigated using a JEOL-JEM 1230 at 80 kV. Images were recorded with a Morada digital camera.

### 2.6 Western blotting

The same two EV enriched pools of ME/CFS patients (N = 5) and healthy controls (N = 5) used for Immuno-TEM were also utilized in Western blot analysis of the generic EV markers CD63, CD9 and TSG101, and the marker albumin (not EV-specific). Two hundred and fifty μl of enriched and pooled samples were lysed using 25 μl of RIPA lysis buffer 10x (20-188, Merck Millipore, Darmstadt, Germany) containing 1% Halt™ Protease and Phosphatase Inhibitor Cocktail (100X) (78440, Thermo Fisher Scientific™, Waltham, MA, USA). Next, 30 μl aliquots of lysed EV enriched sample were mixed with non-reducing 4x Laemmli sample buffer (1610747, BioRad) for CD9 and CD63 detection and with premade 3x sample buffer (1.5 ml 1 M Tris-HCl (pH 6.8), 6.0 ml 10% SDS, 1.0 ml 2% bromophenol blue, 1.5 ml 99% glycerol and 1 ml 1 M DTT) for TSG101 and albumin detection. Samples with the ladder Precision Plus Protein Dual Color Standards (1610374, Bio-Rad) were run on 4–20% Mini-PROTEAN® TGX™ Precast Protein Gels, 10-well, 50 μl (4561094, Bio-Rad) with 1x Tris/Glycin/SDS Buffer (1610732, Bio-Rad). For blotting, a 0.2 μm nitrocellulose membrane (1620112, Bio-Rad) and transfer buffer (1x Tris/Glycine Buffer (1610734, Bio-Rad) with 20% methanol) was used. Blocking was performed using 5% bovine serum albumin (A7906, Sigma-Aldrich) in tris-buffered saline with 0.1% Tween 20 (TBS-T) for CD9 and CD63 detection, and with 5% skimmed milk (70166, Sigma-Aldrich) in TBS-T for TSG101 and albumin detection. Primary antibodies were diluted in TBS-T with 0.02% sodium azide as follows: 1:500 CD63 (10628D, Invitrogen), 1:500 CD9 (10626D, Invitrogen), 1:1000 TSG101 (MA1-23296, Invitrogen) and 1:1000 albumin antibody (sc-271605, Santa Cruz Biotechnology). HRP-conjugated secondary antibody (Anti-mouse IgG, HRP-linked Antibody, 7076, Cell Signaling Technology, Danvers, MA, USA) diluted in 5% skimmed milk in TBS-T as follows: 1:2000 for CD9 and CD63 detection, and 1:1000 for TSG101 and albumin detection. Blots were imaged using ECL™ Prime Western Blotting System (RPN2232, Cytvia) and ImageQuant LAS 4000 (GE healthcare, Chicago, IL, USA).

### 2.7 Single vesicle analysis

ExoView analysis was performed on individual samples from 5 ME/CFS patients and 5 healthy controls by utilizing the EV-TETRA-C chip (NanoView, Malvern, UK) targeting CD63, CD81 and CD9. The technology combines single particle interferometric reflectance imaging sensing with antibody-based capture on a microchip to measure EV size, concentration and the presence of the three tetraspanins on single EVs. The analysis was executed by NanoView, Malvern, UK. In short, EV enriched samples were diluted according to the manufacturers protocol and transferred to microchips with separate wells for each tetraspanin and an anti-mouse IgG isotype control, followed by overnight incubation. Then, the wells were washed three times and incubated with a cocktail of fluorescent antibodies against CD63, CD81 and CD9 before being washed two additional times. Imaging and data acquisition was achieved using the ExoView R100 reader and nScan 2.8.19 software (NanoView). NanoViewer 2.8.10 (NanoView) was used for data analysis with thresholds set to 50-200 nm. All samples were measured in triplicate.

### 2.8 Proteomic analysis

Protein content of all EV enriched samples was analyzed by liquid chromatography tandem mass spectrometry (LC-MS/MS). The LC-MS/MS was performed at the Proteomics Core Facility, University of Oslo and Oslo University Hospital. Protein concentration was measured by BCA assay and 10 μg of protein from each sample was precipitated on MagReSyn amine beads (ReSyn Biosceinces) using 70% acetonitrile. Beads were washed with 100% acetonitrile and 70% ethanol in water and thereafter dissolved in 100 μl 50 mM NH4HCO3 buffer. Proteins were reduced in 10 mM DTT and incubated at 37°C for 30min before alkylation in 15 mM IAA at room temperature in the dark for 30 min 0.5 μg Trypsin (Promega) was added to the samples followed by overnight incubation at 37°C. The resulting peptide mixture was desalted on EVOTIPS according to manufacturer's instructions before analyzed by a nanoLC-MS/MS using EVOSEP coupled to timsTOF fleX (Bruker, Massachusetts, US) with a 15 cm C18 column (EV1137, EVOSEP).

Protein identification and label-free quantitation were done using MaxQuant version 2.4.3.0 [18]. Carbamidomethyl (C) was set as a fixed modification and acetyl (protein N-term), carbamyl (N-term) and oxidation (M) were set as variable modifications. First search peptide tolerance of 20 ppm and main search error 4.5 ppm was used. Trypsin without proline restriction enzyme option was used, with two allowed miscleavages. The minimal unique + razor peptides number was set to 1, and the allowed FDR was 0.01 (1%) for peptide and protein identification. Label-free quantitation (LFQ) was employed with default settings. UniProt database with ‘Human’ entries (2020) was used for database searches.

Further data analysis was performed using the Perseus software version 2.1.1.0 [18]. LFQ intensities were used as input, excluding contaminants, reverse proteins and those only identified by site. The data was log10 transformed and filtered with settings set to a minimum of 50% valid values in at least one group, followed by standard imputation from normal distribution to replace missing values by small numbers representative of the assumed low levels of specific proteins in each sample. Based on low total LFQ intensity, few proteins detected and visual inspection, one sample was considered an outlier and excluded from further analysis resulting in N = 48. The data was z-score normalized before statistical analysis by Mann-Whitney *U* test with *p*-value cut-off = 0.05. FunRich functional enrichment analysis tool version 3.1.3 [19] was used to assess the presence of the identified proteins in the Vesiclepedia database [20] and perform cellular component analysis.

### 2.9 Statistical analysis

The normality distribution of demographic and NTA data was assessed by Shapiro-Wilk test and equality of variances by Levene's test. The non-parametric Mann-Whitney *U* test was used on skewed data with unequal variances while skewed data with unequal variances were analyzed by the Brunner-Munzel test. Normally distributed data with equal variances was analyzed by Student's t-test. A *p*-value <0.05 was considered statistically significant.

## 3 Results

### 3.1 Characteristics of the study phenotypes

The ME/CFS patients and healthy controls included in this study were matched for gender, age (median 31 years) and BMI (Table 1). Most patients reported infection as a disease triggering event (77.3%), and 30.1% were either bedridden or housebound. As many as 29.2% of the ME/CFS patients stated that they have a first degree relative with an autoimmune disease.

**Table 1:** Demographic and clinical characteristics of ME/CFS patients and healthy controls (HC).

	ME/CFS patients	HC	*p*-value
Female [%]^a^	44 [89.8]	45 [90.0]	0.98
Age at inclusion, median [range]^a^	31 [18-58]	31 [18-59]	0.83
BMI, median [range]^b^	23 [17-76]	25 [18-52]	0.25
Disease duration >2 years [%]^c^ ∗	37 [82.2]	-	-
Symptom start following infectious episode, self-reported [ %]^c^	33 [77.3]	-	-
Symptom start following vaccination, self-reported [%]^c^	6 [13.3]	-	-
1st-degree relative with autoimmune disease^d^	14 [29.2]	-	-
Disease severity: [%]^c^ ∗∗			
Bedridden (DSQ79-1)	6 [13.3]	-	-
Strictly housebound (DSQ79-2)	8 [17.8]	-	-
Light housework (DSQ79-3)	21 [46.7]	-	-
Able to work part time (DSQ79-4)	10 [22.2]	-	-

a N = 49 ME/CFS patients and N = 50 healthy controls.

b N = 43 ME/CFS patients and N = 49 healthy controls.^c^ N = 45 ME/CFS patients.^d^ N = 48 ME/CFS patients. ∗Disease duration more than 2 years and/or since childhood/adolescent. ∗∗Disease severity last 6 months prior to inclusion, measured by activity assessment in question no. 79 DePaul Symptom Questionnaire (DSQ79); DSQ79-1) I am not able to work or do anything, and I am bedridden, DSQ79-2) I can walk around the house, but I cannot do light housework, DSQ79-3) I can do light housework, but I cannot work part-time and DSQ79-4) I can only work part time at work or on some family responsibilities.

### 3.2 ME/CFS patients have higher concentrations of EVs than controls

**Fig. 1:**
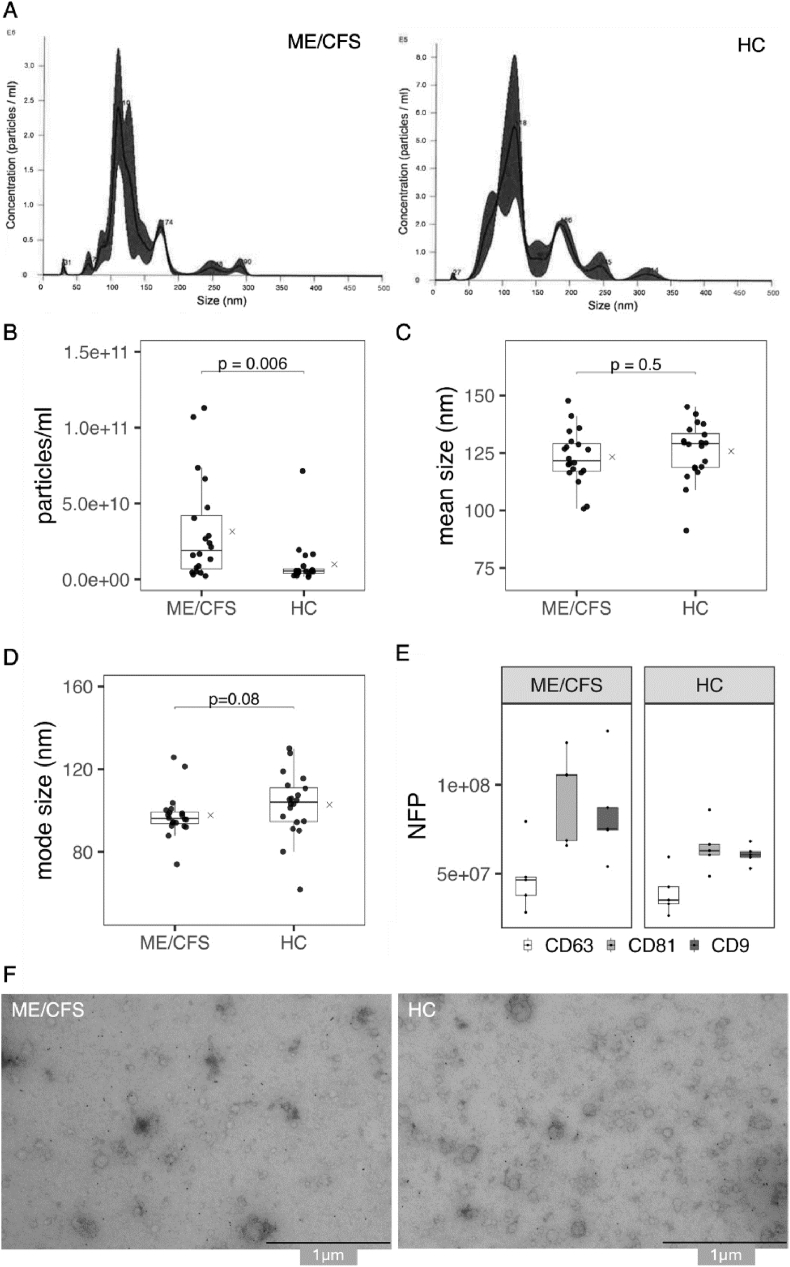
**EV characterization and quantitation** The figures shows the results from randomized and representative ME/CFS patients (N = 20) and healthy controls (HC, N = 20) where a) shows the size distribution measured by NTA, b) shows the EV concentration measured by NTA, c) shows mean size measured by NTA and d) shows mode size measured by NTA and the x represents the group mean in b-d). The ExoView results from randomly selected ME/CFS patients (N = 5) and HC (N = 5) are shown in e) Number of fluorescent particles/ml (NFP) captured by CD63, CD81 and CD9 capture probes using ExoView. For Immuno-transmission electron microscopy (TEM) pools of 5 randomly selected ME/CFS patients and 5 HC were used and is shown in f) CD63-immunoTEM micrographs with 1 μm size bar.

The EV enriched plasma samples were measured by NTA to contain EVs ranging from 50 to 200 nm (Fig. 1a). We observed a significantly higher concentration of EVs in ME/CFS patients compared to healthy controls (*p* = 0.006) (Fig. 1b). The variation in EV concentrations seen among patients did not appear to be reflected in phenotypic heterogeneity, i.e. age, BMI, first-degree relatives with autoimmune disease and self-reported symptom start following infectious episode (Supplementary Fig. 1). The EV sizes were not significantly different, neither measured as mean nor mode, between the ME/CFS patients and controls (Fig. 1c and d).

Next, we characterized the EV populations obtained using our enrichment protocol. TEM micrographs confirmed the EV size estimates, while simultaneously detecting the presence of CD63 positive EVs (Fig. 1f), though only on a small proportion of the EVs. This was in line with the ExoView results, which showed the lowest capture of CD63 positive particles, compared to CD81 and CD9 (Fig. 1e). Additionally, low CD63 concentrations were also confirmed by Western blotting (Supplementary Fig. 2c), likewise the presence of CD9 and TSG101 (Supplementary Fig. 2c) in our EV enriched samples. Higher concentration of albumin, a marker not specific to EVs, was detected in ME/CFS patients compared to healthy controls (Supplementary Fig. 2c). Notably, albumin is present at high levels in blood plasma, both as soluble protein and bound to the ECs as part of the EC protein corona [21], hence the elevated levels could represent plasma protein contamination.

### 3.3 Proteomic analysis shows higher levels of ITIH3 in EVs from ME/CFS patients

Quantitative proteomics was performed on EV samples from 48 ME/CFS patients and 50 controls individually using LC-MS/MS and resulted in 424 reliably identified proteins being included for further analyses. As expected, most of these proteins have been reported to associated with exosomes and extracellular compartments (Supplementary Fig. 2a), and are present in the Vesiclepedia database [20] supporting that our EV enriched was successful (Supplementary Fig. 2b).

**Fig. 2:**
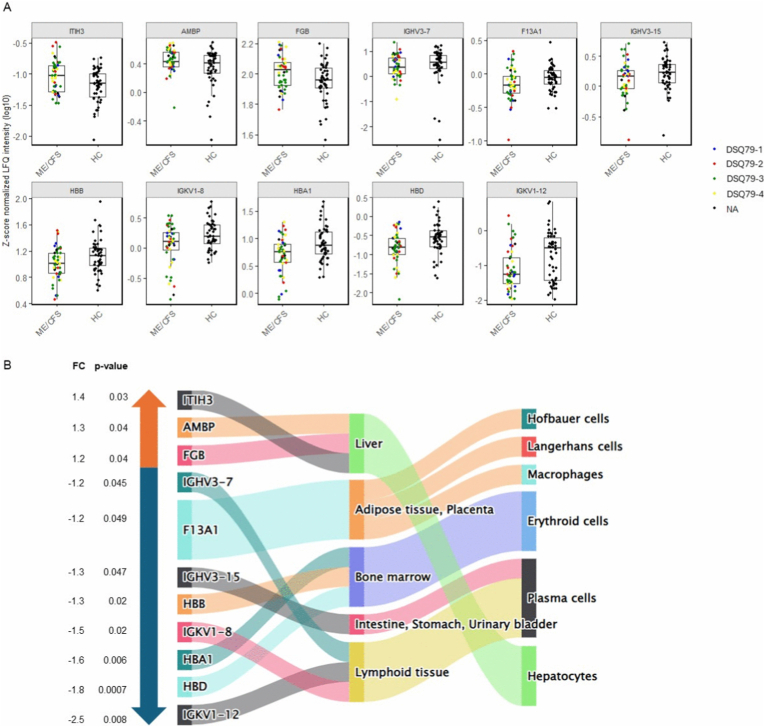
**Differential EV protein levels detected.** a) Detected levels (log10) of EV proteins significantly associated to ME/CFS in cases and controls with disease severity last 6 months prior to inclusion, measured by activity assessment in question no. 79 DePaul Symptom Questionnaire (DSQ79) are colored; blue for DSQ79-1) I am not able to work or do anything, and I am bedridden, red for DSQ79-2) I can walk around the house, but I cannot do light housework, green for DSQ79-3) I can do light housework, but I cannot work part-time and yellow DSQ79-4) I can only work part time at work or on some family responsibilities, black are samples without information (NA, not available), b) predicted cell and tissue origin of ME/CFS associated EV proteins as reported by gene expression across cell and tissue types in the Human Protein Atlas and the proteins' corresponding fold change (FC) and p-values detected in our statistical analysis.

The 178 proteins detected in at least 50% of ME/CFS patients or healthy controls were included in the quantitative analysis of protein levels (Supplementary data). In total, eleven proteins were detected with significantly different levels (p < 0.05) in ME/CFS patients vs healthy controls (Fig. 2, Supplementary Table 1). The EV levels among patients did not appear to correlate with ME/CFS severity. The EV proteins being increased in ME/CFS were Inter-alpha-trypsin inhibitor heavy chain H3 (ITIH3), Alpha-1-microglobulin/bikunin precursor (AMBP) and Fibrinogen beta chain (FGB), while decreased levels were observed for four proteins derived from the variable domains of immunoglobulin light chains (IGKV1-12, IGKV1-8, IGLV3-15 and IGHV3-7), three proteins related to hemoglobin (HBA1, HBB and HBD) and Coagulation factor XIII A chain (F13A1).

To identify the potential cellular and tissue origins of the eleven EV proteins associated with ME/CFS, we analyzed which cells express RNA for these proteins using the Human Protein Atlas ([22], proteinatlas.org) (Fig. 2b–Supplementary Table 1). All the EV proteins increased in ME/CFS patients were liver-specific and expressed by hepatocytes. The EV proteins decreased in patients were mainly expressed by either erythroid cell-specific from the bone marrow or immune system derived by being expressed by plasma B cells.

## 4 Discussion

Herein, we demonstrate that ME/CFS patients have a higher number of EVs present compared to healthy controls. Furthermore, we identified differential abundance of EV proteins associated with ME/CFS. All EV proteins increased in the patients were predicted to originate from hepatocytes, while EV proteins increased in healthy controls appeared to originate from erythroid cells and plasma cells.

Our observation of increased EV levels in ME/CFS is consistent with all previous studies [[7], [8], [9], [10], [11]], despite differences in enrichment methods. Hence, accumulating data clearly points to elevated EV levels in ME/CFS, indicating increased cellular communication via the circulation. Our analyses suggest potential donor cells for these EVs, however, we cannot predict their recipient cells or tissue destination. However, EVs protect their cargo from extracellular degradation enabling them to travel over large distances and even cross the blood brain barrier [23]. Hence, further studies are needed to mechanistically investigate the role of EVs in cellular communication in ME/CFS.

The reports on EV size in ME/CFS are more contradictory. We did not observe any significant differences in EV size, neither did Giloteaux et al. [9] in one study, while they observed larger EVs in ME/CFS patients in another study [11]. Others have suggested that the EVs in ME/CFS are smaller in patients [7,8]. Of note, we observed a slight tendency in the same direction. The discrepancies between studies could be due to technical differences in either EV enrichment or size measurement.

Our results indicated differences in EV protein profiles between the ME/CFS patients and healthy controls. We were unable to replicate the association between ME/CFS and actin networks in EVs by Eguchi et al. [10]. The two studies differed in methodologies applied and sample sizes. Eguchi et al. enriched for EVs by ultracentrifugation and assessed the EV proteome in two small (N = 3) pools of ME/CFS patients diagnosed according to Fukuda criteria [10], while we examined the EV protein profile by size exclusion chromatography of a larger group of ME/CFS patients diagnosed according to the stricter Canadian criteria (N = 48) assessing the proteome individually in each sample. While we found HBA1, HBB and HBD to be reduced in our ME/CFS patients compared to healthy controls, a recent study showed increased levels of HBA, HBB and HBD [24]. These conflicting results could be due to our collection of patients and samples being pre- SARS-CoV-2 pandemic, while their samples were collected from 2020 onwards. Methods used for detection of protein levels (targeted and untargeted) also differ between studies. Even though we have more statistical power than the previously mentioned studies, our differential EV protein levels did not withstand correction for multiple testing indicating that even larger sample sizes are needed in future studies.

Another limitation, which could have influenced our statistical power, is the heterogeneity of the ME/CFS patient group including the exclusion-based diagnosis. We tried to overcome this by using strict diagnostic criteria and investigated the biological heterogeneity in the EV protein levels against clinical variables. Although, we did not see any clear influence on the tested clinical heterogeneity with EV protein levels, future studies, stratifying larger patient cohorts into distinct clinical subgroups might strengthen the reliability of proteomic associations in ME/CFS.

All three EV proteins increased in ME/CFS patients were predicted to mainly originate from hepatocytes. Since our results need to be confirmed in independent cohorts, any interpretation remains speculative, however potentially relevant biological findings may still be discussed. Of note, hepatocytes have been shown to secrete EVs that modify the levels of blood metabolites associated with energy and redox metabolisms, as well as endothelial regulation [25]. ITIH3 is known to be involved in the stabilization of the extracellular matrix, and has been proposed as a biomarker for disease activity in the autoimmune disease myasthenia gravis [26], with elevated ITIH3 levels hypothesized to occur as a response of aberrant complement activation. Interestingly, ME/CFS patients exhibit exercise-associated complement activation, which has been suggested to contribute to inflammation-mediated post-exertional malaise and decreased pain pressure thresholds in patients [27,28]. Recent studies on exercise in ME/CFS patients with sedentary controls, suggests the involvement of the complement and coagulation cascades in both female [14] and male ME/CFS patients [29]. Increased hepatic ITIH3 expression has also been found to reduce mitochondrial respiration in mice [30]. Furthermore, we observed elevated levels of the fibrinogen chains FGB, a crucial member of the coagulation cascade. Giloteaux et al. found that FGA and FGB correlated positively with PEM in patients 24 h post exercise [14]. Among the decreased EV proteins were several immunoglobulin genes which may indicate B cell and antibody alterations. Taken together, the EV proteins increased in ME/CFS patients could have inflammatory roles, particular in the complement and coagulation systems. However, much work remains to link these separate correlations with the complex molecular mechanisms of ME/CFS.

In conclusion, this exploratory study demonstrated significantly elevated levels of plasma EVs in ME/CFS compared to healthy controls. Moreover, several of the putative disease associated EV proteins may be implicated in immune dysregulation, particularly complement and coagulation pathways. To our knowledge, this study represents the largest proteomic EV study in ME/CFS to date, still our study has limitations and is exploratory and warrants further investigation into the role of EVs in the pathophysiology of the disease.

## Authors contributions

Anne Rydland: Writing – review & editing, Writing – original draft, Formal analysis, Data generation and curation, Conceptualization.

Elena Støvring Yran: Writing – review & editing, Data generation and curation, Analysis.

Tuula Anneli Nyman: Writing – review & editing, Data generation and curation.

Elin Bolle Strand: Writing – review & editing, Patient recruitment, Resources.

Anne-Marie Siebke Trøseid: Writing – review & editing, Data generation.

Reidun Øvstebø;: Writing – review & editing, Data generation, Resources.

Fatima Heinicke: Writing – review & editing, Formal analysis, Conceptualization.

Benedicte A. Lie: Writing – review & editing, Writing – original draft, Resources, Formal analysis, Conceptualization.

Marte K. Viken: Writing – review & editing, Writing – original draft, Resources, Formal analysis, Data curation, Conceptualization.

## Declaration of competing interest

The authors declare the following financial interests/personal relationships which may be considered as potential competing interests: Benedicte A. Lie reports financial support was provided by Research Council of Norway. Marte K. Viken reports financial support was provided by The Norwegian ME-association. If there are other authors, they declare that they have no known competing financial interests or personal relationships that could have appeared to influence the work reported in this paper.

## Data Availability

The mass spectrometry proteomics data have been deposited to the ProteomeXchange Consortium via the PRIDE partner repository (https://www.ebi.ac.uk/pride/) [31] with the dataset identifier PXD072203.
