# Supplementary material for: Exploring differences in protein cargo of extracellular vesicles from ME/CFS patient plasma compared to healthy controls
Source: Biochem Biophys Rep. 2026 Jun 20;47:102679. doi: 10.1016/j.bbrep.2026.102679 (PMC13312568; doi:10.1016/j.bbrep.2026.102679)
Supplement: Multimedia component 2 [file mmc2.docx]

**SUPPLEMENTARY TABLE AND FIGURES**

**Supplementary table 1.** EV proteins with significantly altered levels in ME/CFS compared to healthy controls. Tissue and cell types predicted to express these proteins according to the Human Protein Atlas are also included.

| **EV protein** | **Tissue (RNA expression)** | **RNA single cells** | **p-value** | **Fold change** |
| --- | --- | --- | --- | --- |
| ITIH3 | Liver | Hepatocytes | 0.03 | 1.4 |
| AMBP | Liver | Hepatocytes | 0.04 | 1.3 |
| FGB | Liver | Hepatocytes | 0.04 | 1.2 |
| IGHV3-7 | Lymphoid tissue | Plasma cells | 0.045 | -1.2 |
| F13A1 | Adipose tissue, Placenta | Hofbauer cells, Macrophages, Langerhans cells | 0.049 | -1.2 |
| IGHV3-15 | Intestine, Stomach, Urinary bladder | Plasma cells | 0.047 | -1.3 |
| HBB | Bone marrow | Erythroid cells | 0.02 | -1.3 |
| IGKV1-8 | Lymphoid tissue | Plasma cells | 0.02 | -1.5 |
| HBA1 | Bone marrow | Erythroid cells | 0.006 | -1.6 |
| HBD | Bone marrow | Erythroid cells | 0.0007 | -1.8 |
| IGKV1-12 | Lymphoid tissue | Plasma cells | 0.008 | -2.5 |


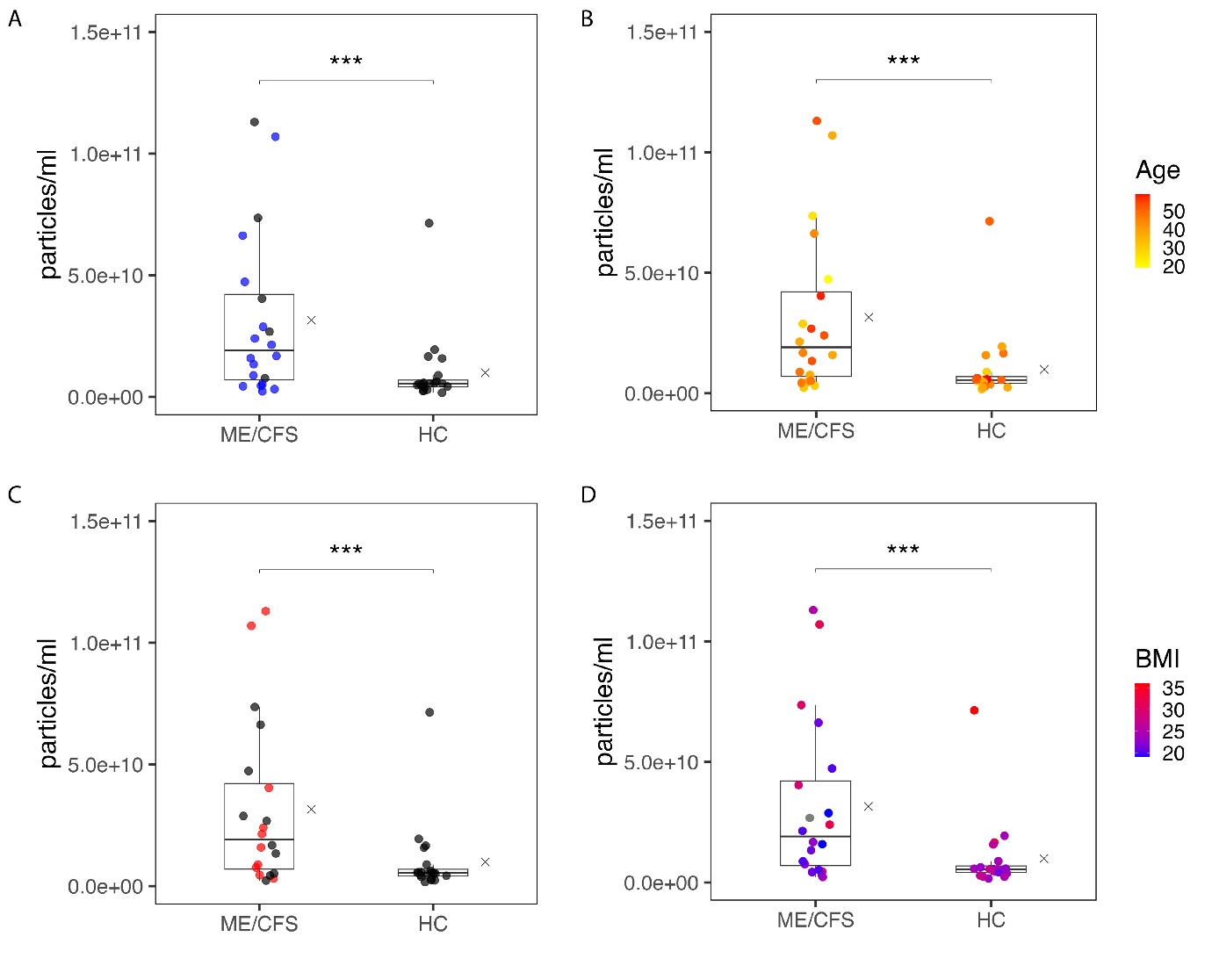


**Supplementary figure 1.** EV concentration in ME/CFS and healthy controls coloured by a) ME/CFS reported to occur following infection (blue), b) age, c) ME/CFS patient with a first degree relative diagnosed with an autoimmune disease (red), d) BMI. ***p-value=0.006.

**
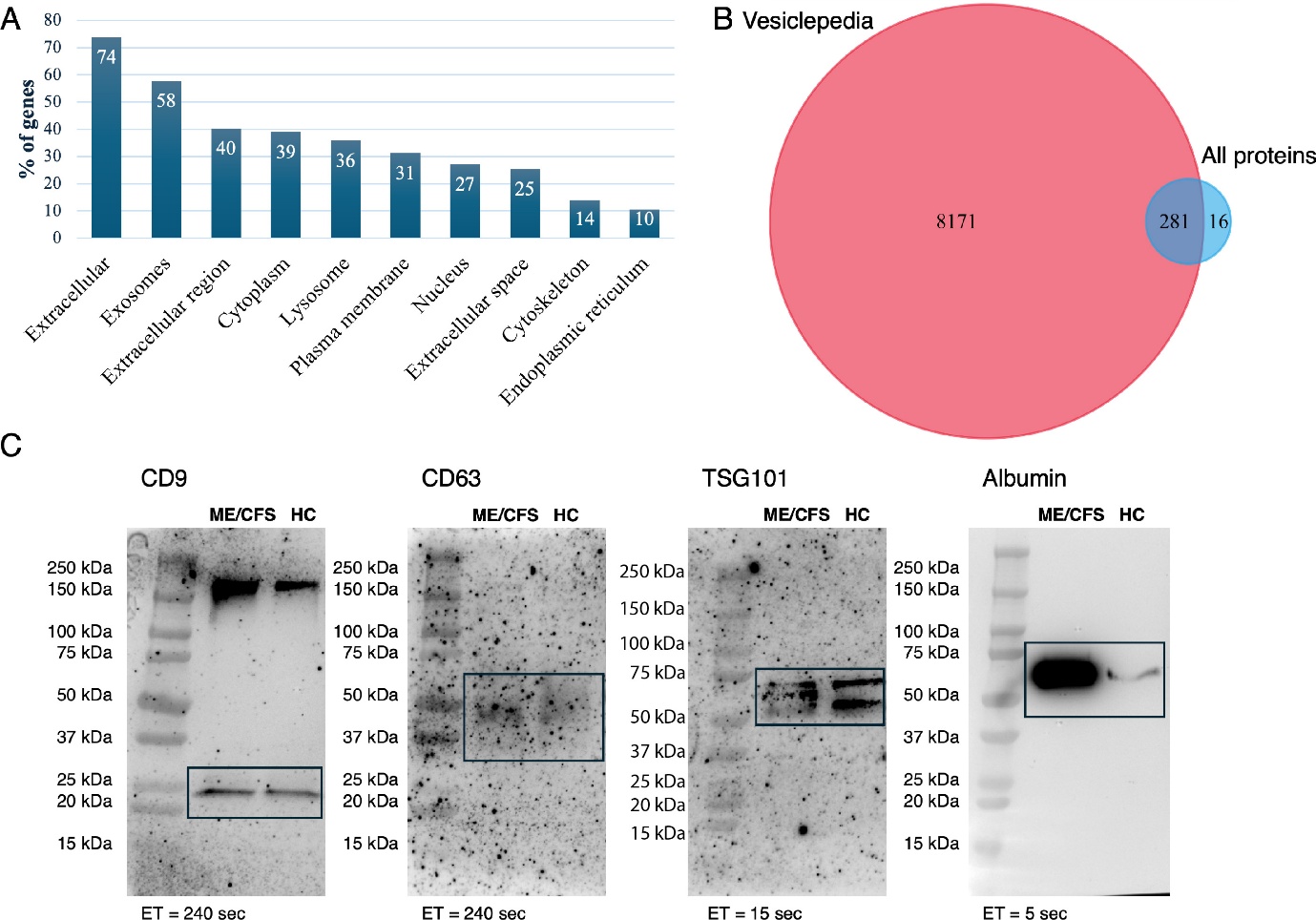
**

**Supplementary figure 2.** Characterization of the reliably detected EV enriched proteins from ME/CFS patients and healthy controls (HC). a) cellular component analysis of the 424 detected proteins performed using FunRich, b) Venn diagram of the 424 detected proteins successfully converted to GeneID in order to compare them to proteins reported in Vesiclepedia, c) western blots detecting the generic EV markers CD9 (~24 kDA), CD63 (between 37-50 kDa) and TSG101 (~46 kDa) and the non-EV marker albumin (~66 kDa) in the two study cohorts. ET=exposure time
